# Supplementary material for: Molecular Typing, Antibiotic Resistance and Enterotoxin Gene Profiles of Staphylococcus aureus Isolated from Humans in South Korea
Source: Microorganisms. 2022 Mar 17;10(3):642. doi: 10.3390/microorganisms10030642 (PMC8952563; doi:10.3390/microorganisms10030642)
Supplement: Supplementary file 1 [file microorganisms-10-00642-s001.zip › microorganisms-1616100-supplementary.pdf]

Table S1. PCR primer pairs used to detect enterotoxin genes in S. aureus strains and their predicted amplification size.

Target gene	Primer Sequence (5′-3′)	Product size (bp)	Reference
sea	GCAGGGAACAGCTTTAGGC<br>GTTCTGTAGAAGTATGAAACACG	520	Monday and Bohach (1999)
seb	ATGTAATTTTGATATTCGCAGTG<br>TGCAGGCATCATATCATACCA	643	
sec	CTTGTATGTATGGAGGAATAACA<br>A<br>TGCAGGCATCATATCATACCA	283	
sed	GTGGTGAAATAGATAGGACTGC<br>ATATGAAGGTGCTCTGTGG	384	
see	TACCAATTAACTTGTGGATAGAC<br>CTCTTTGCACCTTACCGC	170	
seg	CGTCTCCACCTGTTGAAGG<br>CCAAGTGATTGTCTATTGTCG	327	

Reference

28. Monday, SR; Bohach, GA. Use of multiplex PCR to detect classical and newly described pyrogenic toxin genes in Staphylococcal isolates. *Journal of Clinical Microbiology*. 1999, 37(10), 3411-3414.
